# Inter‐ and intraspecific variation in body‐ and genome size in calanoid copepods from temperate and arctic waters

**DOI:** 10.1002/ece3.2302

**Published:** 2016-07-14

**Authors:** Hans Petter Leinaas, Marwa Jalal, Tove M. Gabrielsen, Dag O. Hessen

**Affiliations:** ^1^ Department of BiosciencesUniversity of OsloP.O. Box 1066 BlindernN‐0316OsloNorway; ^2^ The University Centre in SvalbardP.O.Box 1569171LongyearbyenNorway

**Keywords:** Bergmann clines, *Calanus*, climate zones, *flow cytometry*, *Paraeuchaeta*

## Abstract

The tendency of ectotherms to get larger in the cold (Bergmann clines) has potentially great implications for individual performance and food web dynamics. The mechanistic drivers of this trend are not well understood, however. One fundamental question is to which extent variation in body size is attributed to variation in cell size, which again is related to genome size. In this study, we analyzed body and genome size in four species of marine calanoid copepods, *Calanus finmarchicus*,* C. glacialis*,* C. hyperboreus* and *Paraeuchaeta norvegica*, with populations from both south Norwegian fjords and the High Arctic. The *Calanus* species showed typical interspecific Bergmann clines, and we assessed whether they also displayed similar intraspecific variations—and if correlation between genome size and body size differed between species. There were considerable inter‐ as well as intraspecific variations in body size and genome size, with the northernmost populations having the largest values of both variables within each species. Positive intraspecific relationships suggest a functional link between body and genome size, although its adaptiveness has not been settled. Impact of additional drivers like phylogeny or specific adaptations, however, was suggested by striking divergences in body size – genome size ratios among species. Thus, *C. glacialis* and *C. hyperboreus*, had fairly similar genome size despite very different body size, while *P. norvegica,* of similar body size as *C. hyperboreus,* had the largest genome sizes ever recorded from copepods. The inter‐ and intraspecific latitudinal body size clines suggest that climate change may have major impact on body size composition of keystone species in marine planktonic food webs.

## Introduction

Body size is linked to fitness in numerous ways. Several indirect effects may be important, such a sexual selection, predation and resource availability. However, central in life history theory are the direct effects of body size on fecundity on one hand, and on time and energy requirement needed to reach maturity on the other (Stearns 1992), and consequently the trade‐off between size and age at maturity (Roff 1981; Stearns and Koella 1986). One aspect of this trade‐off that has received much attention is the relationship between adult body size and temperature (Hessen et al. 2013).

A trend of increasing body size along geographical gradients with decreasing temperature, originally described for endotherms (Bergmann 1847), has also been documented for many ectotherms; that is Bergmann clines (Atkinson 1994; Angilletta et al. 2004a). Although widespread, the trend is not universal. In particular terrestrial ectotherms give many examples of the opposite trend of animals being smaller in the cold, defined as converse Bergmann cline (Mousseau 1997). Among marine ectotherms, however, Bergmann clines seem quite common (Timofeev 2001; Rees et al. 2007, 2008; Hessen and Persson 2009), and thus offer many opportunities to study this phenomenon in different taxa. Causations for Bergmann clines appear quite complex, however, and may vary among species (Watt et al. 2010; Forster et al. 2012; Hessen et al. 2013). It will partly represent phenotypic plasticity, with animals tending to reach larger size at maturity with decreasing developmental temperatures, but also reflect genotypic differences (Atkinson 1994; Angilletta et al. 2004b). Such temperature effects have implications for climate change. In addition to affecting individual performance, changing body size of keystone species could also seriously affect marine productivity and food webs (Daufresne et al. 2009). Because of these potentially important consequences, it is of great interest also to improve our understanding of the underlying drivers affecting the phenomena, both at the individual as well as cellular level.

Variation in body size among organisms is determined either by the number of cells, the size of cells, or a combination of both (Kozłowski et al. 2003; Hessen et al. 2013). In this context, cell size is more convenient to analyze than cell number, even though large‐scale analyses of cell size in metazoans are not straightforward. Instead, genome size may serve as a useful proxy for cell size. A strong positive correlation between genome size and cell size appears rather universal in both plants and animals (Cavalier‐Smith 1978; Bennett 1987; Gregory et al. 2000; Gregory 2005). The usefulness of this correlation is that genome size can be analyzed on large samples with good accuracy by use of flow cytometry (FCM) (Jeffery and Gregory 2014).

Marine crustaceans show many examples of increasing body size with decreasing water temperature (e.g., Timofeev 2001; Rees et al. 2007, 2008). This has been particularily well doccumented among copepods species (Falk‐Petersen et al. 2009; Hessen and Persson 2009; Parent et al. 2011; Lin et al. 2013). Calanoid copepods represent a diverse and abundant group, with many key species in marine ecosystems (Bron et al. 2011). Some species have wide latitudinal distribution, others show sequential replacement of related species along thermal gradients with different degree of overlap (Thibault et al. 1999; Broms et al. 2009; Reygondeau and Beaugrand 2011).

Calanoid copepods typically have large genomes relative to other planktonic crustaceans (Gregory et al. 2000; Hessen and Persson 2009), but with great variations among species (McLaren et al. 1988; Wyngaard et al. 2005; Bron et al. 2011). In general, the genome size appears positively correlated with body size (McLaren et al. 1988; Gregory et al. 2000; Hessen and Persson 2009), suggesting that differences in body size between copepod species at least partially reflect variation in cell size in line with the nucleocytoplasmic hypothesis (Cavalier‐Smith 1978). This is supported by the observation that some Copepoda of similar stages also have corresponding cell numbers, meaning that the difference in body size is related to cell volume (McLaren and Marcogliese 1983). It is also likely that genome size is related to developmental rate (Escribano et al. 1992). The occurrence of related calanoid species with wide and partly overlapping ranges offer opportunities to study the relation between body‐ and genome size in a climate perspective at lower taxonomic levels in more detail.

To improve our understanding of causations and mechanisms underlying the temperature‐related trends in body and genome size, analyses should include con‐specific populations from contrasting climates and comparisons of variation within and between species. In such approaches, one must also take into account other factors that may covary with temperature along body size clines.

In this study, we selected four marine calanoid copepods of different body size, which occurred both in two fjords of southern Norway and in the high arctic waters of Svalbard. These were the three closely related *Calanus* species *C. hyperboreus* Kroyer*, C. glacialis* Jaschnov and *C. finmarchicus* (Gunnerus), and the genetically more distant predatory species *Paraeuchaeta norvegica* (Boeck). The *Calanus* species differ greatly in body size and latitudinal range, showing the typical patterns of an interspecific Bergmann cline (e.g., Broms et al. 2009; Parent et al. 2011; Hessen et al. 2013). In this study, we take advantage of the two predominantly arctic species *C. glacialis* and *C. hyperboreus* having marked disjunctive distribution patterns with isolated populations in some fjords of southern Norway. In addition, Svalbard and the southern Norwegian fjords represent quite extreme points in the geographical rage of *C. finmarchicus* (Broms et al. 2009). These populations were included to elucidate possible thermal responses also on this important keystone species of the North Atlantic pelagic food web. The last species, the predatory *P. norvegica,* is widely distributed in the North Atlantic and arctic waters (Auel 2004) and was included in the study as a genetically distant species with very different ecology from the herbivorous *Calanus* species. More specifically we wanted to test (1) if variation in body size between arctic and temperate populations of each species shows similar patterns as previously described by interspecific Bergmann clines of the *Calanus* species, and (2) whether correlation between genome size and body size shows a consistent pattern across species or intraspecific differences.

## Materials and Methods

### Study organisms

The three focal *Calanus* species differ distinctly in size (see Table 1). The smallest of them, *C. finmarchicus*, is the most southern one, with main distribution in the North Atlantic, but partly extending into arctic waters (Blaxter et al. 1998; Hirche and Kosobokova 2007). In Oslofjorden, it is less abundant than the more southern relative *C. helgolandicus* (Claus). The latter species was only found in the southern sites, and therefore not a part of this study. The medium sized *C. glacialis* is mainly associated with arctic shelf seas, although occasionally being observed in the Norwegian sea (Mauchline 1998; Broms et al. 2009). It is, however, numerically dominant in Lurefjorden about 30 km north of Bergen. The much larger *C. hyperboreus* has its main distribution in the Arctic basins (Mauchline 1998). It is scarce in the Norwegian Sea, apparently without successful reproduction (Broms et al. 2009), but does exist as isolated populations in deeper parts of some of the southern Norwegian fjords such as Oslofjorden. The last and more distantly related species, *P. norvegica*, has about the same maximum body size as *C. hyperboreus*. It is common in southern Norway as well as in arctic waters. Several other *Paraeuchaeta* species larger than *P. norvegica*, occur in the Arctic, but with no known occurrence in southern Norway.

**Table 1:** Body length (cephalothorax) variations between temperate and high arctic con‐specific populations of four calanoid copepods

Population	Area	Location	Sampling month	*n*	Mean	SD	95% C.I.
*Calanus*							
*C. helgolandicus*							
Oslofjorden	Southern fjord	59°19′N; 10°35′E	Apr‐15	19	2.35	0.09	2.31–2.39
*C. finmarchicus*							
Oslofjorden	Southern fjord	59°19′N; 10°35′E	Apr‐15	14	2.54	0.09	2.49–2.59
Fram Strait	High Arctic	79°05′N; 08°01′E	Jul‐13	5	2.80	0.10	2.74–2.85
Kongsfjorden	High Arctic	78°58′N; 11°51′E	Jan‐14	25	2.71	0.12	2.66–2.76
*C. glacialis*							
Lurefjorden	Southern fjord	60°41′N; 5°8′E	Aug‐11	14	2.56	0.10	2.50–2.62
Billefjorden	High Arctic	78°39′N; 16°44′E	Dec‐11	11	3.40	0.20	3.26–3.53
Billefjorden	High Arctic	78°39′N; 16°44′E	Oct‐12	11	3.37	0.12	3.29–3.44
Billefjorden	High Arctic	78°39′N; 16°44′E	Mar‐13	21	3.38	0.17	3.30–3.46
Rijpfjorden	High Arctic	80°30′N; 22°25′E	Jan‐12	13	3.40	0.16	3.30–3.49
*C. hyperboreus*							
Oslofjorden	Southern fjord	59°19′N; 10°35′E	Feb‐13	24	5.04	0.60	4.78–5.29
Hinlopen	High Arctic	79°38′N; 18°51′E	Sep‐12	19	6.85	0.77	6.50–7.22
Fram Strait	High Arctic	79°08′N; 8°02′W	Apr‐13	11	6.61	0.19	6.48–6.74
*Paraeuchaeta*							
*P. norvegica*							
Oslofjorden	Southern fjord	59°19′N; 10°35′E	Feb‐13	13	5.35	0.22	5.22–5.48
Lurefjorden	Southern fjord	60°41′N; 5°8′E	Jun‐13	6	5.58	0.13	5.44–5.71
Fram Strait	High Arctic	79°05′N; 08°01′E	Jul‐13	12	6.13	0.60	5.77–6.49

### Sampling

**Figure 1:**
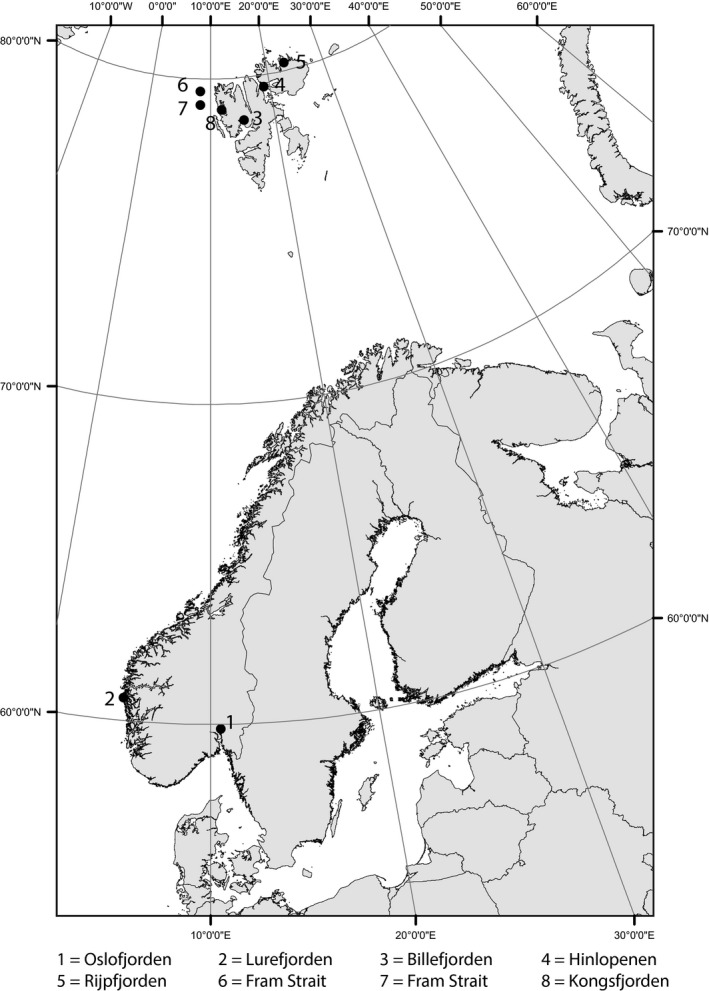
Locations of the two southern and six northern sites where calanoid copepods were sampled for this study.

The complete list of locations and time of sampling of each species as well as sample size are given in Table 1, and the location of the sites are shown in Figure 1. The southern sites consisted of Oslofjorden and Lurefjorden, which differ fundamentally in predation regime. Lurefjorden is completely dominated by nonvisual invertebrate predators, such as the jellyfish *Periphylla periphylla,* and only a few visual predators (Bagøien et al. 2001; Sørnes et al. 2007), while visual predators, such as sprat, are common in Oslofjorden (Solberg et al. 2015). The arctic samples were taken from altogether six sites of the Svalbard archipelago (Fig. 1), depending on from where we were able to sample arctic material of the different species. To evaluate possible temporal variation within a population, we sampled *C. glacialis* at one of the arctic site (Billefjorden) once each year from December 2011 to March 2013.

Samples were taken at all locations by use of WP2 nets with 0.5 m diameter and 200 *μ*m mesh size (Gabrielsen et al. 2012), or with WP3 nets with 1 m2 opening and 1000 *μ*m mesh size. From most sites samples had to be shipped for several days to our laboratory (Oslo). For the flow cytometer analyses, we needed living animals, and to improve survival, densities had to be kept low during transport and thereby reducing the possible sample size. After picking out the adults from each sample three to six animals were selected for genome size analyzes (see below), and body length and species identification were performed on the rest of the sample.

### Species identification

In field studies, separation of the *Calanus* species is often based on size, although some overlap in body size may occur (Parent et al. 2011; Gabrielsen et al. 2012). To verify species identification, the samples were identified genetically, and the few samples where genetic analysis showed a mixture of our focal *Calanus* species were excluded from the study, as we then could not be sure that genome size had been analyzed on a homogenous one‐species sample.

The *Calanus* species were identified based on their restriction patterns after digesting amplicons of a partial sequence of their mitochondrial 16S rDNA with DdeI and VspI according to Lindeque et al. (1999). DNA was extracted using the E.Z.N.A. Tissue DNA kit (Omega Biotek, Norcross, GA, USA Inc) and PCR, restrictions and separation of digested amplicons were performed according to Gabrielsen et al. (2012). Representative individuals from all taxa were sequenced to ensure the correct interpretation of the digestion patterns, and unique sequences among these were submitted to Gen Bank (accession numbers KX371797‐KX371807).

Identification of *P. norvegica* was performed by sequencing the mtDNA cytochrome c oxidase I (COI) barcoding region amplified by the LCO1490 and HCO2198 primers (Folmer et al. 1994). DNA was extracted from 23 individuals of the three analyzed *Paraeuchaeta* populations as described above. PCR was run on an Eppendorf Eppendorf Mastercycler (ep gradient S) in 25 *μ*L volumes including 1× DreamTax buffer, 0.125 U DreamTaq, 0.2 *μ*mol/L of each primer, 0.125 *μ*g BSA, and 200 *μ*mol/L of each of the nucleotides. The cycling conditions were: 95°C—3, 10× (95°C—30″, 46°C—30″, 72°C—60″), 25× (95°C—30″, 50°C—30″, 72°C—60″), 72°C—7′, 15°C—hold. The positive PCR amplicons were cleaned using the E.Z.N.A. Cycle Pure Kit (Omega Bio‐Tek Inc) and Sanger sequenced at GATC Biotech AB, Constance, Germany using the LCO1498 primer. Unique sequences were submitted to Gen Bank (accession numbers KX371791‐KX371796) An alignment of our six unique *Paraeuchaeta* sequences and the 12 *Paraeuchaeta* COI sequences available in Gen Bank by December 2015 (AB379996, AB380008, AF531748‐49, AY660600‐02, FJ602478, FJ602502‐03, KC287809‐10) was assembled using MUSCLE (Edgar 2004) in Seaview v. 4.5.5. Maximum likelihood analysis was run based on a GTR model with heterogeneous sites using PHYML in Seaview v. 4.5.5. Our *Paraeuchaeta* sequences were all placed in a well‐supported clade (1000 bootstrap resamplings) with the available *P. norvegica* sequences.

### Body size analysis

Body size was measured as the length of cephalothorax of living adults laying on their sides, in a small plastic well (Ø = 16 mm) with seawater just covering their bodies. All measurements were carried out using a digital Leica DFC 425 camera on a Leica M205C microscope. The images were analyzed by Leica application suite (LAS version 3.7.0; Leica Microsystems, Wetxlar, Germany). We focused on adult size, as this is a life history trait generally assumed to be closely linked to fitness (e.g., Stearns 1992). This choice reduced the amount of animals available for the analyses compared with the common use of the much more abundant last copepodit stage (CV). The only exception to this procedure was arctic *C. finmarchicus*, of which we were unable to get adult individuals in altogether four field cruises. Because of its key role in the North Atlantic, we nevertheless included arctic samples of this species by using CV, in order to exploit a possible increase in body size in arctic waters also in this species. This would not lead to erroneous arguments about a relative increase in size in northern populations, only a moderate underestimation of the increase (see e.g., Madsen et al. 2001). Similarly, a comparison of body length of 24 adults and 58 CV of arctic *C. glacialis* from the same two samples showed only a relatively small difference in size between these two last instars (mean ± SE: adults = 3.4 mm ± 0.07; CV = 3.2 mm ± 0.04) (*F*_1,80_ = 10.97, *P* = 0.001).

Significant size differences between samples were tested by general linear models, comparing mean and 95% C.I. of each sample. This and box plot of body length of arctic versus temperate animals of each *Calanus* species were performed in JMP 9 (SAS Institute, Cary, NC). To estimate relative difference in body length between animals from the southern and northern sites in each species, all con‐specific data from each climate zones were pooled and fitted to log‐linear models with a Gaussian likelihood. Relative differences with confidence intervals were then computed directly as the exponent of linear contrasts from the models in R3.1.2 (R Development Core Team 2014).

### Genome size analysis

**Figure 2:**
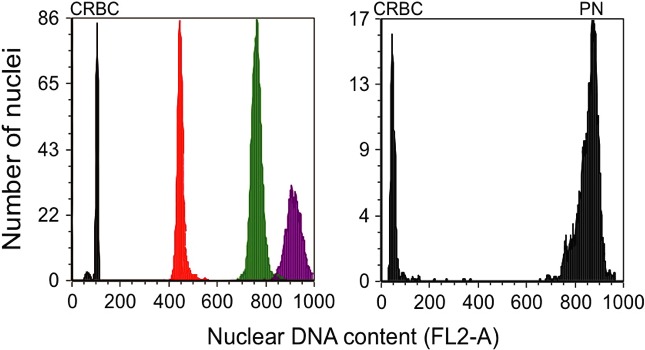
Representative flow cytometer DNA histograms of a selected population of each copepod species, compared with the standard chicken red blood cells (CRBC); *Calanus finmarchicus* (red), *Calanus glacialis* (green),and *Calanus hyperboreus* (purple). *Paraeuchaeta norvegica* (PN) is represented by a separate histogram because a different FL2‐A voltage was applied than for the other copepods because of its large genome. FSC voltage was same for all. *Note also that in these selected populations there was no overlap in genome size between C. glacialis and C. hyperboreus*. Other populations showed considerable overlap, as shown in Table 2.

Genome size was analyzed on whole individuals by FCM, basically following the protocol of Jalal et al. (2013). In brief, animals were ground in grinding buffer (Korpelainen et al. 1997), followed by incubation with 1 mg RNase A (Invitrogen Life Science, Waltham, MA) and 50 *μ*g propidium iodide (PI; Invitrogen Life Science, Waltham, Mass. USA). Chicken fresh blood cells (CRBC) of *Gallus gallus domesticus* (5.0 × 10^5^ cells/mL) were used as internal standard. The standard DNA‐content of CRBC was set to 2.5 pg DNA (Vergilino et al. 2009). In addition to CRBC nuclei, 2.5 *μ*m alignment beads (Invitrogen Life Science) were used to keep instrument settings (amplification and sample rate) constant throughout the experiment and to confirm low coefficient of variation alignment. Depending on size, three to six adult copepods were analyzed from each sample, which was sufficient to obtain reliable and highly repeatable counts in succeeding analyses. Animals from all populations were analyzed for genome size except for *C. finmarchicus* from Oslofjorden where the analysis failed to stain properly for unknown reasons. The genome size (DNA‐content) was gated and measured with high precision by FCM, as illustrated by the distinct peaks in Figure 2. Stained samples were analyzed with a FACS Calibur flow cytometer equipped with a 488 nm laser (Becton, Dickinson, NJ). The PI fluorescence emission signal was measured in a FL2 detector (585/42 bandpass filter setup). Doublets and cell aggregates were discriminated from the analysis by gating around the singlet population in fluorescence pulse width (FL2‐W) versus pulse area (FL2‐A) dual parameter cytogram (Shapiro 2003). *Paraeuchaeta* was analyzed with a different FL2‐A voltage than the other copepods because of its large genome, but we then also used the same chicken blood cells as internal standard, so this will not bias the results. We did however also run a comparative analysis with the same voltage for *C. hyperboreus* and *Paraeuchaeta*. The FCM results were analyzed using FCS express 3 (De Novo, Los Angeles, CA) and Modfit LT software (Verity, Topsham, ME). Calculation of nuclear DNA‐content was made according to Galbraith et al. (2001).

## Results

**Figure 3:**
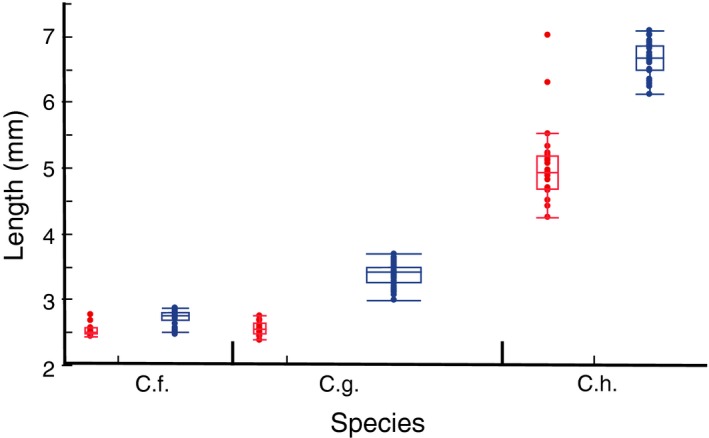
Box plots of median length of the three *Calanus* species from their temperate (red) and arctic (blue) sampling sites. C.f.: *Calanus finmarchicus*; C.g.: *Calanus glacialis*; C. g.: *Calanus hyperboreus*. Width of boxes scales with samples sizes. Horizontal extensions of boxes and lines represents the 25% and 75% quantiles, respectively. Observations outside this range are represented by dots.

The arctic populations of the three *Calanus* species showed the same rank in body size as previously described from their respective main areas; that is *C. finmarchicus* being smallest and *C. hyperboreus* largest (Fig. 3; blue box plots). They all differed significantly, with nonoverlapping 95% C.I. (Table 1). Because measurements of the arctic *C. finmarchicus* was made on the CV stage, a direct comparison with the adult arctic *C. glacialis* should be performed with caution. However, this size difference was strongly supported by an additional analysis showing that these CV of *C. finmarchicus* to be significantly smaller than CVs of arctic *C. glacialis* (3.2 ± 0.04 mm) (ANOVA: *F*_1,86_ = 52.0; *P* < 0.0001). Among the southern *Calanus* populations *C. hyperboreus* was by far the largest species, but there were no significant difference between *C. finmarchicus* from Oslofjorden and *C. glacialis* from Lurefjorden (Table 1). In fact, even the CV of the arctic *C. finmarchicus* was significantly larger than the adults of the temperate *C. glacialis* from Lurefjorden (Table 1).

In addition, a fourth *Calanus* species, *C. helgolandicus*, co‐occurred with *C. finmarchicus* in the samples from Oslofjorden, where in constituted 58% of the adults analyzed. This southern *Calanus* species was smaller than the co‐occurring *C. finmarchicus*, with nonoverlpping 95% C.I. (Table 1), which fits the overall general pattern of decreasing body size of species with successively more southern range.

In all four focal species, the arctic populations were significantly larger in body sizes than the temperate ones (Table 1). But, by far the largest relative differences were seen in the two primarily arctic species *C. glacialis* and *C. hyperboreus*, with the Svalbard animals being respectively 32.3% (95% C.I.: 27.2–37.5%) and 34.7% (95% C.I.: 30.0–39.5%) longer than the southern population. There were no differences between the arctic populations of *C. glacialis* from Billefjorden and Rijpfjorden, and samplings from Billefjorden for three succeeding years gave virtually identical results (Table 1). Similarly, we found no significant difference between the two arctic populations of *C. hyperboreus* (Table 1). In *C. finmarchicus,* the arctic CV was 7.2% (95% C.I.: 2.8–11.8%) longer than the southern adults. Also *P. norvegica* was moderately larger in arctic waters, with the adults being on average 12.8% (95% C.I.: 2.8–18.3%) longer than the southern animals. The two southern populations of the species did not differ significantly (Table 1).

**Figure 4:**
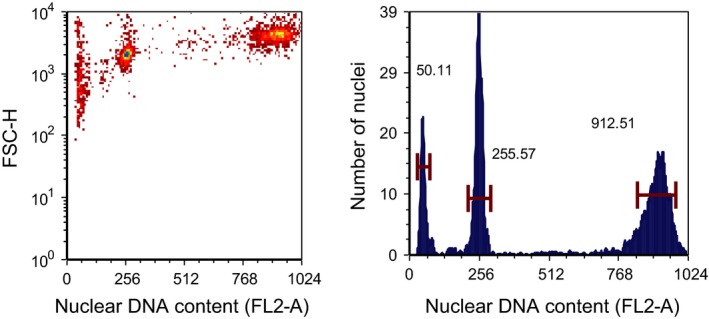
Flow cytometer gating outputs (left panel) of *Calanus hyperboreus* and *Paraeuchaeta norvegica* analyzed from the same sample (with the same FL2.A voltage). FSC‐H = forward light scatter. Left peak is the standard (chicken red blood cells), *C. hyperboreus* in the middle and *P. norvegica* to the right. Corresponding histograms in right panel.

The genome size varied from <6 pg haploid DNA/cell in *C. finmarchicus* to >32 pg haploid DNA/cell in *P. norvegica* (Table 2). All three species that were analyzed from both arctic and southern sites display a pronounced intraspecific variability. In both *C. glacialis* and *C. hyperboreus,* the arctic populations had distinctly larger genomes than those from the south (Table 2). In *P. norvegica,* on the other hand, the population of Oslofjorden had substantially smaller genome than both the other temperate population (Lurefjorden) and the arctic population (Table 2). The genome size of *P. norvegica* is truly astonishing, with the maximum size being nearly three times larger than previously recorded maximum for calanoids. To get a full resolution for genome size of *Paraeuchaeta*, these were analyzed with a different FL2‐A voltage, but for direct comparison we also made an analysis with *C. hyperboreaus* and *P. norvegica* in the same sample and with the same voltage, clearly demonstrating the striking difference in genome size (Fig. 4).

**Table 2:** Genome size variations between temperate and high arctic con‐specific populations of four calanoid copepods

Population	Area	Mean	SD
*Calanus*			
*C. finmarchicus*			
Fram Strait	High Arctic	5.48	0.02
*C. glacialis*			
Lurefjorden	Southern fjord	8.45	0.04
Billefjorden	High Arctic	11.08	0.36
Billefjorden	High Arctic	10.20	0.20
Billefjorden	High Arctic	11.11	0.32
Rijpfjorden	High Arctic	11.29	0.02
*C. hyperboreus*			
Oslofjorden	Southern fjord	9.20	0.07
Hinlopen	High Arctic	10.55	0.13
Fram Strait	High Arctic	11.48	0.33
*Paraeuchaeta*			
*P. norvegica*	High Arctic		
Oslofjorden	Southern fjord	23.09	0.12
Lurefjorden	Southern fjord	32.75	1.57
Fram Strait	High Arctic	32.23	0.08

**Figure 5:**
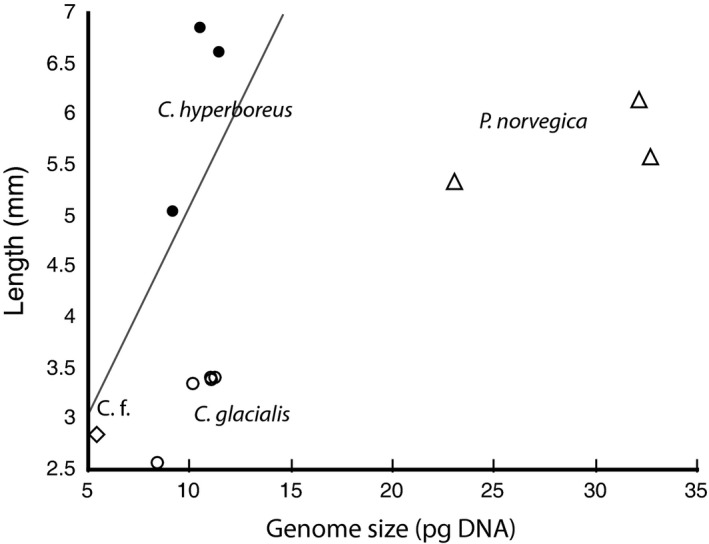
Relationships between body length and genome size (*C*‐value) in populations of the species in Table 1. A linear regression for length over genome from a large number calanoid copepod marine and freshwater species (*P* < 0.001, *r* ^2^ = 0.72, *n* = 29; from Hessen and Persson 2009) is included for comparison with our results.

Each of the three species with multiple populations analyzed for both body length and genome size showed a positive relationship between the two variables (Fig. 5). In both *C. glacialis* and *C. hyperboreus,* body size increased strongly with genome size, and much more so than seen in *P. norvegica*. However, the data‐plot of two of the species diverged markedly from the genome size – body length regression previously obtained from 29 species of copepods (see Fig. 5). In particular, *P. norvegica* had much larger genome size relative to body size. Also *C. glacialis* differed quite clearly from the general genome size – body length regression line for copepods, with notably larger genome size relative to body length than expected from the average norm. In both *C. glacialis* and *C. hyperboreus,* body size increased strongly with genome size, and much more so than seen in *P. norvegica* (Fig. 5).

## Discussion

The three *Calanus* species represent a typical example of an interspecific Bergmann cline, although considerable variation in size within species has been described (Parent et al. 2011; Gabrielsen et al. 2012). Here we show a consistent intraspecific trend in that each of these species, as well as *P. norvegica,* were all significantly larger in arctic compared with temperate waters. This may at least partly reflect phenotypic plasticity, as increasing body size with decreasing developmental temperature is a general phenomenon in ectotherms (e.g., Angilletta et al. 2004b; Walters and Hassall 2006). However, size at maturity trade‐off with other important life history traits, such as fecundity and age at maturity, and may have great impact on vital rates (Stearns 1992). This suggests that the temperature effects may have selective consequences, depending on the environment, and thus that also genotypic differences are involved. The great variation among species in the degree of size differentiation between southern and arctic populations may indicate differential selection among species. In any case, the results emphasize that climate change may affect the size structure of marine food webs both by favoring smaller species (Daufresne et al. 2009), and by reducing the body size of important keystone species, such as the dominant calanoid copepods.

Previous studies from Lurefjorden and adjacent waters have shown *C. finmarchicus* to be significantly smaller than *C. glacialis* in this area (Eiane et al. 1999; Niehoff and Hirche 2005). In contrast to this, we found no size difference between *C. glacialis* from Lurefjorden and *C. finmarchicus* from Oslofjorden. Consequently, in these two locations *C. finmarchicus* appears smallest where it coexists with high abundance of the larger *C. glacialis*, and larger where the somewhat smaller *C. helgolandicus* is numerically dominant. This difference might reflect ecological character displacement possibly driven by competitive interactions (e.g., Dayan and Simberloff 2005), but an evaluation of his idea would require a more detailed study.

The southern populations of *C. glacialis* and *C. hyperboreus* have probably been isolated from their main arctic populations for a very long time, which would facilitate evolutionary adaptations to their contrasting environments. *Calanus finmarchicus* and *P. norvegica* have more widespread distribution (Longhurst 2007), with populations in arctic waters more likely being influenced by gene flow from Atlantic waters (Astthorsson and Gislason 2003; Parent et al. 2011), which might tend to counteract evolutionary adaptation to cold water. Consistent with this line of reasoning, the arctic and southern populations of the two latter species differed less in body size, possibly more influenced by phenotypic plasticity. Nevertheless, the shallow sill of the threshold‐ fjords of the south may to varying degree represent barrier to gene flow between resident inner fjord populations and oceanic con‐specifics. This may result in local adaptations as argued above for the apparent body size differences between *C. finmarchicus* from Oslofjorden and Lurefjorden. However, to explore these questions in more detail, phenotypic plasticity versus genotypic differences could be studied by comparing thermal reaction norms for body size between conspecific populations in common garden experiments.

Temperature is generally explained as the main causation for Bergmann clines, either as direct effects on developmental rates or metabolism, or indirect effects via for example food availability or oxygen solubility (Verbek et al. 2011). However, other factors may also be involved. In copepods, visual predation is a well‐known driver for reduced body size, and Kaartvedt (2000) related the dominance of larger *Calanus* species at high latitude to reduced importance of visual predators in these waters. Similarly, low visual predation in the turbid waters of Lurefjorden has been assumed to favor *C. glacialis* over the smaller *C. finmarchicus* there (Eiane et al. 2002). However, the great difference in size of *C. glacialis* between Lurefjorden and the Arctic, despite low visual predation at both sites, does indicate a predominantly thermal effect on body size.

Similar evidence for temperature effects is seen in *P. norvegica,* with the two southern populations having the same relatively small size, in spite of very different predation regimes in the two fjords. Likewise, the great size difference between arctic and temperate *C. hyperboreus*, also suggests a temperature effect. However, in this case additional impact of abundant visual predators in the Oslofjorden cannot be ruled out. Taken together, we argue that these data give strong support to temperature as a main driver of size differences between southern and arctic populations of these calanoid species, but that trophic interactions (competition and predation) and indirectly also oxygen concentrations (Verbek et al. 2011) and physical properties of the water column may pose additional effects.

A positive correlation between genome size and body size is commonly reported in invertebrates (Ferrari and Rai 1989; Finston et al. 1995) including calanoid copepods (McLaren et al. 1988; Gregory et al. 2000; Hessen and Persson 2009). This, together with a generally positive correlations between genome size and cell size (Cavalier‐Smith 1978; McLaren and Marcogliese 1983; Gregory 2005) indicate that differences in body size between related species at least partly reflects changes in cell size (Kozłowski et al. 2003; Hessen et al. 2013). However, our results emphasize that a small subset of species may diverge substantially from the general picture. Considerable variability in genome size among species of similar body size was seen in arctic *C. finmarchicus* versus temperate *C. glacialis,* and *C. hyperboreus* versus *P. norvegica*.

There was also little difference between *C. glacialis* and *C. hyperboreus* despite their great difference in body size, reflecting disproportionately larger genomes in *C. glacialis* than expected from the general body ‐ and genome size regression line for copepods (Hessen and Persson 2009). The causation for this is not known, but differences in life cycle and growth patterns might play a role. Irrespective of causation, the very large genome of *P. norvegica* is truly remarkable. Within Crustacea this is only matched by some observations from cold‐water Arctic amphipods (Rees et al. 2008). This species belongs to a well‐defined family (Park 1994) and thus is genetically distant from other calanoid copepods. Consequently, the large genome size may reflect phylogeny (cf. Gregory 2005), but *Paraeuchaeta* also possesses striking morphological modifications, such as appendages and sensory organs, which in itself may have a linkage to genome size (Gregory et al. 2000).

In contrast to the complex pattern of species differences, intraspecific variations were much more consistent, with distinctly positive correlation between genome and body size, suggesting some general response mechanisms underlying the intraspecific Bergmann clines. Thus both phylogeny and trait specific constraints should be taken into account in order to elucidate the complexity of the mechanistic coupling between genome and body size in relation to climate.

## Concluding Remarks

All four calanoid species of this study showed inter population differences in body size consistent with intraspecific Bergmann clines. The previously recorded strong correlation between body‐ and genome size in copepods was more or less masked by interspecific differences in our data, comparing this limited set of species. However, within each species, the relationship was much clearer, suggesting that on this level intraspecific differences in body size may largely depend on changes in cell size. On a longer time scale, however, macro‐evolution may act on cell size in a lineage specific way, leading to body size differences gradually becoming more dependent on cell number.

Our results also point to some fundamental questions that should be addressed in future studies for better understanding basic processes underlying the observed patterns. (1) While body size may be greatly affected by phenotypic plasticity, the genome size more directly points to genotypic differences. Although our results consequently suggest micro evolutionary changes within each species, such an argument needs to be tested more directly by common garden experiments comparing conspecific populations at different temperatures. (2) Large genome size in cold waters has been linked to slower growth and metabolic rates at reduced temperatures, as large genomes generally go along with low growth or developmental rates (Escribano et al. 1992; Kozłowski et al. 2003; Gregory 2005). The causality, however, is not settled. We do not yet know whether selection for larger individuals promotes larger genomes or vice versa. In fact, the causality may work both ways, and the effect of temperature might represent direct selection for large genomes in the cold, or simply reduced selection against large genomes at low temperature and low growth rates (Hessen et al. 2013).

## Conflict of Interest

None declared.
